# Loss to follow-up and associated factors among adult people living with HIV at public health facilities in Wakiso district, Uganda: a retrospective cohort study

**DOI:** 10.1186/s12913-019-4474-6

**Published:** 2019-09-04

**Authors:** Denis Opio, Fred C. Semitala, Alex Kakeeto, Emmanuel Sendaula, Paul Okimat, Brenda Nakafeero, Joaniter I. Nankabirwa, Charles Karamagi, Joan N. Kalyango

**Affiliations:** 1 Clinical Epidemiology Unit, Makerere University College of Health Sciences, P.O. Box 7072, Kampala, Uganda; 2 Makerere University- Johns Hopkins University Research Collaboration (MU-JHU), P.O. Box 23491, Kampala, Uganda; 3 Department of Internal Medicine, Makerere University College of Health Sciences, P.O. Box 7072, Kampala, Uganda; 4 Makerere University Joint AIDS Program (MJAP), P.O. Box 7072, Kampala, Uganda; 5 Infectious Diseases Research Collaboration (IDRC), P.O. Box 7475, Kampala, Uganda; 6 Institute of Public Health and Management, Clarke International University, P.O. Box 7782, Kampala, Uganda; 7 Department of Paediatrics and Child Health, Makerere University College of Health Sciences, P.O. Box 7072, Kampala, Uganda; 8 Department of Pharmacy, Makerere University College of Health Sciences, P.O. Box 7072, Kampala, Uganda

**Keywords:** Loss to follow-up, HIV care, PLHIV, Public health facilities

## Abstract

**Background:**

Loss to follow-up (LTFU) from care among people living with HIV (PLHIV) is thought to be more common in the public setting compared to the private health care. It is anticipated that the problem may become worse with the current “test and treat” policy in Uganda due to the likely increases in patient loads and its attendant pressure on health care providers to support patient counseling. This study determined the incidence and factors associated with LTFU from HIV care among adult PLHIV in public health facilities in Wakiso district, Uganda.

**Methods:**

This was a retrospective cohort study that involved the review of 646 records of patients initiated on antiretroviral therapy (ART) between January 1st, 2015 and December 31st, 2017 at 13 randomly selected public health facilities in Wakiso district. The cox proportional hazards regression was used to determine the factors associated with LTFU. The results were supported by sequential in-depth and key informant interviews to explore reasons for LTFU.

**Results:**

Of the 646 patients enrolled, 391 were female (60.5%), 282 were below 30 years (43.6%) and 207 were married (50.1%). A total of 216 patients (33.4%) had no documented outcomes and were considered LTFU. The incidence of LTFU was 21 per 1000 person months (95% confidence interval (CI): 18–25 per 1000 person months). Factors associated with LTFU included having normal weight compared to underweight (adjusted hazard ratio (aHR) 0.64, 95% CI: 0.45–0.90, p = 0.011), receiving HIV care from hospitals compared to lower level facilities (aHR 0.22, 95% CI: 0.12–0.41, p < 0.001), and no telephone contact compared to those with a telephone contact (aHR 2.16, 95% CI: 1.33–3.51, p = 0.002). Stigmatization and long waiting times were the prominent reasons for LTFU reported from the in-depth and key informant interviews.

**Conclusions:**

The incidence of LTFU in public health facilities in Uganda is quite high and is associated with being underweight, not having a telephone contact to receive reminders and receiving care at lower level facilities. Early diagnosis, routine use of patient address locator forms and improved quality of HIV care at lower level health facilities may reduce LTFU among PLHIV.

**Electronic supplementary material:**

The online version of this article (10.1186/s12913-019-4474-6) contains supplementary material, which is available to authorized users.

## Background

The Human Immunodeficiency Virus/Acquired Immune Deficiency Syndrome (HIV/AIDS) epidemic remains a major problem especially in countries of sub-Saharan Africa like Uganda [1]. The prevalence of HIV among adults aged 15 to 64 was estimated at 6.2% in 2017, which corresponds to an estimated 1.2 million people living with HIV (PLHIV) in the country [2]. More importantly, it was estimated that 26,000 Ugandans died of AIDS-related illnesses in 2017 [3]. The available means of management and control of HIV include treatment with antiretroviral therapy (ART), post-exposure prophylaxis (PEP) and pre-exposure prophylaxis (PrEP) [4]. When effective ART is used, it decreases the risk of transmitting the HIV virus from one person to another [4, 5], and allows for the HIV positive person to live a healthy life [5]. Patient retention in care is crucial to ensure ongoing receipt of ART, timely evaluation of ART toxicity and/or new opportunistic infections; these in turn help to reduce HIV related morbidity, mortality, the incidence of new infections, and development of ART resistance [5–9].

Despite the benefits of retention in care, it is challenging for the health care facilities (HCFs) to keep all patients in HIV-care [10]. Studies in HIV centres of excellence in Uganda have shown the incidence of loss to follow-up (LTFU) from HIV care to range from 9 to 20% [11, 12]. It is hypothesized that the incidence may be higher in public facilities, where patients do not receive the extra support (e.g. provision of transport refund and meals on follow-up visit dates) provided by the centres of excellence (model institutions that provide high quality of comprehensive care for PLHIV, and are often funded by international agencies). This may further be augmented by the “test and treat” policy (where populations at risk are screened for HIV infection, and diagnosed HIV infected individuals receive immediate treatment regardless of their CD4 cell count or clinical stage) since patients are likely to be many and not very ill during ART initiation. Such patients may not perceive themselves to be at risk of the complications of HIV, and it may therefore be more difficult to retain them in care, until such a time as when their immune function has gone down and they present with complications [13]. Although the problem of LTFU in the centres of excellence has been widely evaluated, the burden in public facilities where resources are constrained, and yet a larger load of patients are received, is not well appreciated [10, 14]. We assessed the incidence of, and the factors associated with LTFU from HIV care among adult patients enrolled at public facilities in Wakiso district, Central Uganda, between January 2015 and December 2017.

## Methods

### Study setting

The study was conducted in Wakiso district, Central Uganda which neighbors the country’s Capital City, Kampala. The district consists of 12 divisions, six sub-counties and 720 villages with an estimated population of 2,111,061 people [15]. The district has the highest prevalence of HIV in Uganda at 10% [16]. It has 31 public HCFs including; one hospital at district level, six health centre IVs (HCIVs) at Health Sub District (HSD) level and 24 health centre IIIs (HCIIIs) at sub-county level. All these facilities are supported by the government of Uganda, have HIV clinics, and provide HIV care at no charge to the patient [16]. Each HIV clinic is headed by the Doctor, who is supported by a Clinical Officer (provides diagnosis and treatment), Counsellors (counsel patients on adherence and other psychosocial needs), a data management team (manage the ART clinic database) and volunteers (organize the patient files). The HIV clinics are provided with technical support by Mildmay, Uganda, a non-governmental organization that supports government efforts toward HIV/AIDS response. The study was conducted in the hospital (Entebbe Hospital), all the six HCIVs in Wakiso district (Wakiso, Namayumba, Kasangati, Buwambo, Ndejje, and Kajjansi) and six randomly selected HCIIIs (Kira, Nabweru, Nsangi, Kakiri, Nakawuka, and Kigungu). In 2015, the proportion of people who tested positive out of the total number tested was: 551 out of 9628 at the hospital, 2525 out of 32,677 at the HCIVs, and 1429 out of 21,366 at the selected HCIIIs [16]. The study involved data abstraction in the period of ART initiation based on CD4 cell count (January 01st, 2015 to December 31st, 2016) and ART initiation based on the “test and treat” treatment strategy (January 01st, 2017 to December 31st, 2017).

### Study population

There are 31 public HCFs in the study area including one hospital, six HCIVs and 24 lower level facilities (Health Centre IIIs). For this study, 13 facilities including the hospital, all six HCIVs and six HCIIIs were selected for the study. The general hospital and all the six HC IVs were included in the study because they are referral centres for the lower HCFs. Simple random sampling was used to select six out of the 24 HCIIIs (one HC III from each of the six HSDs). Probability proportional to size sampling was used to determine the number of patients that were selected at each HCF. Finally, systematic sampling was used to select the eligible patients from each facility to include in the study.

All files for HIV positive patients aged 18 years and above and initiated on ART between January 2015 and December 2017 at the selected facilities were screened for eligibility to be included in the study. Patient files were excluded if they missed data on important variables including date for start of ART, 1st line regimen, sex, age and CD4 cell count at initiation.

### Study design

The study used quantitative and qualitative data collection methods. For quantitative data collection, a retrospective review of participants’ medical records was used to collect information on: participants’ demographics, date of ART initiation, baseline CD4 cell count, clinical status at initiation, appointment dates, patient status by December, 2017, as well as level of health facility attended (Hospital or HCIV or HCIII).

For qualitative data collection, key informant interviews (KIIs) and in-depth interviews were conducted to explore the reasons for LTFU from HIV-care. The data collected included: distance from home to health facility, economic reasons, capacity of the ART clinics, stigma, waiting time at the clinic, spiritual and cultural beliefs, and conduct of the clinic staff. The KIIs involved three doctors and one expert client selected from ART clinics with the highest incidence of LTFU. Participants in the KIIs were selected using purposive sampling based on their knowledge and role in management of HIV patients. The in-depth interviews involved patients receiving care from the selected HCFs. The patients’ homes were traced, using the telephone contact extracted from the ART cards, to conduct the interviews. Saturation was reached after conducting nine in-depth interviews (three with patients LTFU and six with active patients). The KIIs were conducted by the Principal Investigator whereas the in-depth interviews were conducted by Research Assistants. All interviews were conducted using an interview guide developed for this study (See Additional file 1 and Additional file 2), and were recorded using an audio recorder.

### Data management and analysis

The definition of LTFU in this study was adopted from the Uganda Ministry of Health definition which is “a patient who has not visited the health facility HIV clinic in three or more consecutive months at any point in their care since they initiated ART” [17]. The incidence of LTFU was defined as “the number of patients missing clinic visits for three or more consecutive months divided by the total follow-up time”.

The Cochran method was used to calculate the sample size for estimation of incidence of LTFU for the study [18]. The parameters used in this sample size estimation included: the standard normal (Z) value corresponding to 95% confidence interval, level of precision of +/− 5%, and an estimated retention level of 70% [19]. The sample size estimated by this method (*N* = 323) was inflated by a design effect of two to cater for clustering in LTFU at health facility level giving total sample size of 646 [20]. Sample sizes for the Logrank test and the cox proportional hazard regression model were estimated using the Lachin method [21]. The parameters used in the sample size calculation for Logrank and cox model included: the Z- value corresponding to 5% level of significance for a two-sided test, Z- value corresponding to 80% power of study, hazard among patients with CD4 count<250cells/mm^3^ of In (0.68) and hazard among patients with CD4 count≥250cells/mm^3^ of In (0.32) [11]. The estimated sample size (N = 176) was inflated by a design effect of two giving 352, as the minimum number of required participants [20]. The bigger sample size of 646 was used since it was sufficient to cater for all objectives.

All data were extracted into a data abstraction form and entered into Epidata Version 3.1, and were exported to STATA Corp version 13.1 for analysis. The cox proportional hazards regression was used to determine the factors associated with LTFU. The times were censored for patients who either died or transferred to other ART clinics. Factors with P-value of less than 0.2 at bivariate analysis, were considered for multivariate analysis. The estimates were adjusted for clustering at the different HCF levels. Interaction and confounding were assessed for in the regression model. Associations were presented as hazard ratios and P-values of less than 0.05 were considered statistically significant.

Logrank test was conducted to compare the retention times of patients enrolled on the basis of CD4 cell count (January 01st, 2016 to December 31st, 2016) and those enrolled on the basis of “test and treat” (January 01st, 2017 to December 31st, 2017). The Kaplan Meier curve was used to graphically compare the retention times of the two groups.

Information from the in-depth interviews was collected in Luganda and translated into English language before transcription; whereas KIIs were only transcribed into text form. The information was coded and synthesized using Open Code version 4.02 to generate prominent themes, then thematic analysis was done.

## Results

### Description of the study subjects

**Fig. 1:**
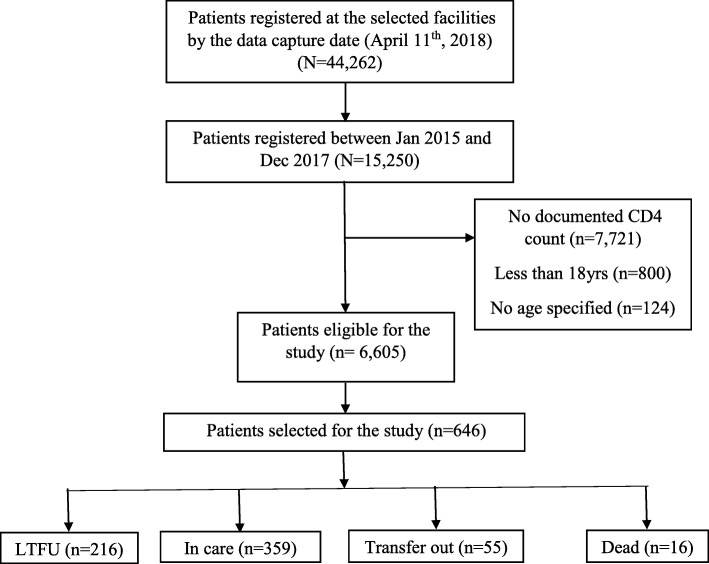
A flow diagram describing the patients included in the study and their various outcomes

A total of 44,262 patients were ever registered at the 13 selected HIV clinics by December 2017, of which 15,250 (34.4%) were initiated on ART between January 2015 and December 2017. Of the 15,250 patients initiated on ART, 8645 (56.7%) did not meet the eligibility criteria as detailed in (Fig. 1). A random sample of 646 patients was selected from the 6605 eligible patients to join the study. Majority of patients in this study were female (60.5%) and 282 (43.6%) aged < 30 years. A total of 122 (29.5%) patients were not married and 177 (29.8%) had no telephone contacts documented on their ART cards. Most of the patients (82.8%) had a baseline CD4 count less than 500c/μl and majority (89.7%) were in WHO clinical stage I or II. Nearly all the patients (98.3%) were initiated on Tenofovir/Lamivudine/Efavirenz as the initial ART regimen. Table 1 provides the details of the demographic characteristics of the study population.

**Table 1:** Baseline demographic and clinical characteristics of 646 patients included in study, Wakiso district, Central Uganda cohort, January 2015–December 2017

Variable	N	(%)	Incidence Rate (per 1000 persons)	95% CI
Sex of subject				
Male	255	(39.5)	18	14–24
Female	391	(60.5)	23	19–29
Age group				
Below 30 yrs.	282	(43.6)	32	26–40
30- 44 yrs	268	(41.5)	16	12–21
45 yrs. and above	96	(14.9)	13	8–21
Marital status (*n* = 413)				
Never married	122	(29.5)	19	13–29
Married	207	(50.1)	21	16–28
Living together	18	(4.4)	11	1–23
Divorced/Separated	44	(10.7)	15	6–42
Widowed	22	(5.3)	28	17–48
Telephone contact (*n* = 595)				
Yes	418	(70.2)	16	13–21
No	177	(29.8)	33	26–42
CD4 cell count				
Less than 500c/ml	535	(82.8)	21	17–25
500c/ml or more	111	(17.2)	27	18–40
WHO clinical stage (*n* = 639)				
I	359	(56.2)	21	17–27
II	214	(33.5)	21	16–27
III	54	(8.4)	29	17–49
IV	12	(1.9)	11	2–87
BMI (*n* = 449)				
Underweight	68	(15.1)	20	12–36
Normal weight	312	(69.5)	15	12–20
Over weight	52	(11.6)	27	16–46
Obese	17	(3.8)	24	10–61
Regimen				
TDF/3TC/EFV	635	(98.3)	21	18–24
Others^a^	11	(1.7)	95	36–252
Care entry (*n* = 554)				
Out patient	430	(77.6)	22	18–27
eMTCT	74	(13.4)	27	17–42
Others^b^	50	(9.0)	23	13–41
Health facility level				
Hospital^c^	100	(15.5)	13	9–20
HCIV^d^	432	(66.9)	21	17–26
HCIII^e^	114	(17.6)	25	18–36

CI- Confidence Interval; Others^a^- AZT/3TC/NVP & TDF/3TC/NVP; Others^b^- Inpatient, Outreach, STI, TB & Transfer-In; ^c^- Hospital level had 69.7% case records completeness; ^d^- HCIV had 72.1% case records completeness; ^e^- HCIII had 78.6% case records completeness

### Incidence of LTFU

**Fig. 2:**
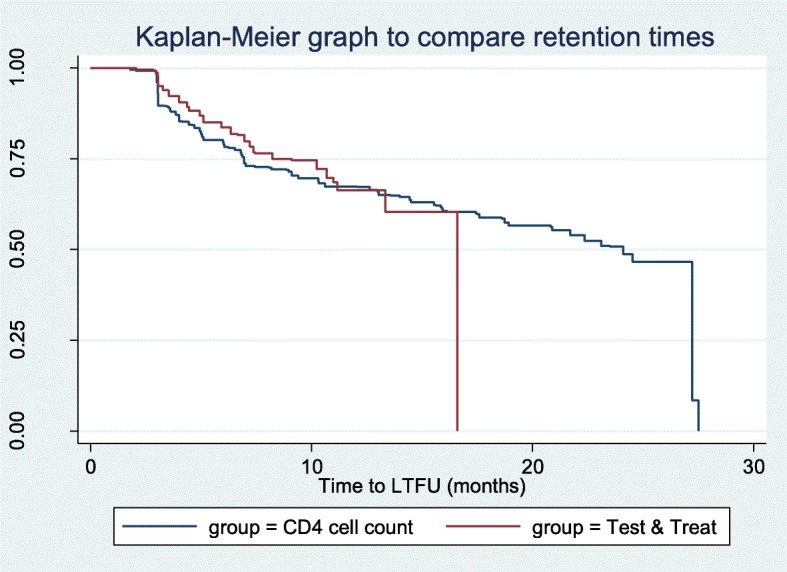
Kaplan-Meier graph comparing the time to LTFU of patients initiated on basis of ‘CD4 cell count’ and ‘Test and Treat’ strategy

By the end of December 2017 only 359 of the 646 (55.6%) patients were still in care at the health facility where they were initiated on ART. A total of 55 (8.5%) patients were transferred out of the HIV clinic where they were initiated on ART, 16 (2.5%) patients died while 216 (33.4%) patients were lost to follow-up (Fig. 1). The incidence rate of LTFU was 21 per 1000 person months (95% Confidence Interval (CI): 18–25 per 1000 person months). Female patients had a higher incidence of LTFU (23 per 1000 person months) compared to the males (18 per 1000 person months). Patients aged less than 30 years had a higher incidence of LTFU (32 per 1000 person months) compared to patients aged between 30 and 44 years (16 per 1000 person months) and patients 45 years and above (13 per 1000 person months) (Table 1). The median retention time of patients enrolled on the basis of CD4 cell count (January 01st, 2016 to December 31st, 2016) was 24.1 months while that of patients enrolled on the basis of “test & treat” (January 01st, 2017 to December 31st, 2017) was 16.6 months (Fig. 2). Patients initiated with a CD4 count of 500c/μl and above had a greater incidence rate of LTFU (27 per 1000 person months) compared to those with CD4 count of less than 500c/μl (21 per 1000 person months) (Table 1).

### Factors associated with LTFU

The adjusted cox regression model suggested that factors- normal weight compared to underweight (adjusted hazard ratio (aHR) = 0.64, 95%CI: 0.45–0.90), no documented telephone contact (aHR = 2.16, 95%CI: 1.33–3.51), and hospital level compared to health centre III level (aHR = 0.22, 95%CI: 0.12–0.41) were significantly associated with LTFU (Table 2). The hazard ratios for age (aHR = 0.99, 95%CI: 0.98–1.00) and hospital level (aHR = 0.97, 95%CI: 0.94–0.98) varied significantly over time.

**Table 2:** Cox regression model to determine factors associated with LTFU, Wakiso district, Central Uganda cohort, January 2015–December 2017

	Univariate				Multivariate		
Characteristic	HR	95%CI	*P*-value		aHR	95%CI	*P*-value
Sex							
Male	Ref*						
Female	1.28	1.03–1.59	0.028				
Age	0.96	0.95–0.98	<0.001		0.98	0.95–1.01	0.116
Age#time	0.99	0.98–1.00	<0.001		0.99	0.98–1.00	0.032
Marital status							
Never married	Ref*						
Married	0.97	0.69–1.36	0.857				
Living together	0.91	0.40–2.10	0.830				
Divorced/Separated	0.70	0.30–1.63	0.405				
Widowed	1.37	0.66–2.87	0.399				
BMI							
Under weight	Ref*				Ref*		
Normal weight	0.68	0.48–0.96	0.027		0.64	0.45–0.90	0.011
Over weight	1.01	0.55–1.87	0.965		0.90	0.43–1.91	0.789
Obese	1.18	0.59–2.33	0.644		0.99	0.52–1.88	0.981
Telephone contact							
Yes	Ref*				Ref*		
No	2.15	1.59–2.90	<0.001		2.16	1.33–3.51	0.002
CD4 count							
Less than 500c/ml	Ref*						
500c/ml and above	1.29	0.85–1.94	0.228				
WHO clinical stage							
I	Ref*						
II	0.94	0.65–1.36	0.733				
III	1.07	0.72–1.60	0.741				
IV	0.62	0.31–1.25	0.181				
Health facility level							
HC III	Ref*				Ref*		
HC IV	0.77	0.53–1.12	0.166		0.58	0.28–1.23	0.156
Hospital	0.51	0.39–0.68	<0.001		0.22	0.12–0.41	<0.001
Hospital#time	0.95	0.94–0.96	<0.001		0.97	0.94–0.98	0.001
Care entry							
Others*	Ref*						
Out patient	1.03	0.67–1.60	0.879				
eMTCT	1.24	0.63–2.46	0.535				

HR- Hazard Ratio; aHR- Adjusted Hazard Ratio; Ref*-Reference category; Others*- Inpatient, Outreach, STI, TB & Transfer-In; #- Interaction

### Reasons for LTFU from the qualitative research

#### Capacity of the ART clinics

The capacity to retain patients in care in most clinics was very limited, mainly due to the overwhelming number of patients that visited these clinics. One Key Informant (KI) disclosed: *“But then also we have only one counsellor and yet we have so many clients. So, the time they take counselling these clients is very minimal. Sometimes we do it in group and yet it should be done on an individual basis, because many people have different backgrounds and issues to deal with…sometimes there are certain issues you can talk about and somebody remembers something but then in a group setting, many times it’s hard.”*

#### Stigma

Stigma has contributed to a large number of patients missing their appointments in the clinic. A patient revealed in an in-depth interview “*…….she really looks at me like I killed a human being. It makes me feel terribly bad. I am over stigmatized in my family.”* The problem of stigma was also prominent during KIIs. One KI disclosed *“And sometimes, we have clients who do not want to be transferred to a facility nearer to where they are living, yet they can’t foot the bill (transportation) when we need them. I think it is stigma issues.”* The problem was also echoed by a different KI *“…….stigma is still high. People don’t want to be seen coming from the ART clinic. Instead of coming to collect medicine in time, they find themselves staying because they don’t want to be seen*.”

#### Distance to health facility and economic reasons

The findings also indicated that some patients had to move a long distance from home to the health facility for drug replenishment. On days that they don’t have money, they would get to miss clinic appointments. A KI reported that *“Others, the economic factors, loss of a job, or loss of bread winner in the family (especially women).*” “...*Then the other issue is that some of them come from far places. Sometimes, transport becomes a challenge, and they stay back….”* (Another KI disclosed). A patient reported that “……*So, one time I asked her (sister) to send me some transport money, she started to hurl insults at me. She said you should tell your men who infected you to give you transport.”*

#### Waiting time at the clinic

Long waiting time at the clinic was experienced by majority of the patients. This was majorly because of low capacity to serve the many patients that are being managed at these clinics. A KI narrated that *“Then, the other issue is that in government we have huge turn-ups. And yet some do not have time to wait. One may want to come early, get a service and then proceed. So, even when they come early, they will find a line, and then they just go. So, they decide to leave just because the line is long.”*

#### Spiritual and cultural beliefs

The findings also suggested that some patients relied on spiritual and cultural beliefs for cure. *“Some patients get misled, and they abandon treatment…others seek spiritual healing and after they have been prayed for; they say they have healed; they don’t come back again.”* (One KI disclosed).

#### Conduct of the clinic staff

The manner in which the clinic staff handle the patients is also very important. Some patients found the staff unprofessional in the clinics. A patient said *“……you will find that some of the staff are young girl students, who do not know how to handle people like us.”* Another patient said *“…...they back-bite us and on many occasions they are gossiping….and some of them are very arrogant”.*

The prominent reasons for LTFU from both the in-depth interviews and KIIs were stigma, capacity at the health facilities and long waiting time.

## Discussion

This study sought to determine the incidence of, and associated factors with LTFU in public health facilities of Wakiso district, Central Uganda. Of the 646 patients enrolled in the study period, more than half of the patients were still in care, close to 10% of the patients were transferred out, 2.5% of the patients died, while a third of the patients were lost to follow-up. The incidence of LTFU was 21 per 1000 person months. Underweight at the time of initiating ART, lack of a documented telephone contact, and receiving HIV care from lower level health facilities compared to a hospital –based HIV clinic were significantly associated with LTFU. Surprisingly, there was no significant (p = 0.231) mean difference in the retention times of patients initiated on ART based on CD4 cell count compared to those initiated under the “test and treat” strategy.

Over 69,498.2 total person months, we found the incidence of LTFU of 21 per 1000 person months. Meaning, every month, 21 out of 1000 persons were LTFU in the ART clinics. This rate was much higher than for studies conducted in centres of excellence in Uganda, which have shown an incidence between 1.3 per 1000 person months to 4.1 per 1000 person months [11, 22]. The difference in incidence between the two (public service delivery and centres of excellence) could be explained by the fact that centres of excellence offer extra support to patients. The extra support in care includes provision of transport refund for some of the visits and meals on follow-up visit dates, which are not available in public health facilities [23]. In addition, public facilities are often overcrowded, with limited number of health care providers and long waiting periods which is unattractive for patients [24]. The difference in estimates may also be due to the difference in the definition of LTFU used; for instance, a study by Okoboi et al. (2015) reported an incidence rate of LTFU at 1.59 per 100 person years, equivalent to 1 per 1000 persons every month that was a value much lower than what we observed in our study. However, the study defined LTFU as failure to visit centre at least once in 6 months which reduces the number in the numerator and thus a lower incidence [11].

In this study (Fig. 2) we found that patients initiated on ART on the basis of CD4 cell count were more likely to be retained in care than those initiated based on “test and treat” strategy, although the comparison was not statistically significant (*p* = 0.231). The increased risk of LTFU observed could be due to the fact that patients initiated based on “test and treat” strategy have been in care for a shorter time compared to those initiated based on CD4 cell count. However, the difference may also be attributed to the fact that patients initiated based on “test and treat” strategy are often not very ill and may not perceive themselves to be at risk of the complications of HIV, hence this may make it more difficult to retain them in care. In addition, the conduct of the health workers as revealed in the KIIs could have also contributed to the increased LTFU in the patients enrolled on a “test and treat” basis. However, other studies have linked early ART initiation to a decreased risk of LTFU [25, 26].

Factors associated with LTFU included: not having a documented telephone contact, getting care from a lower health facility and being underweight. Appointment reminders and home follow-ups of patients when present at a care facility are often via a phone call [27]. Our study reported that patients with no documented telephone contacts were twice as likely to get LTFU as those with documented telephone contacts. This means that patients without documented telephone contact would miss clinic appointment reminders, and thus miss clinic visits. This finding was similar to that found in the M-track study where sending mobile message reminders was associated with an increased likelihood of keeping clinic appointments [28].

Normal weight was found to be associated with a decreased risk for LTFU, compared to underweight, in our study. The normal weight patients were 36% less likely to get LTFU compared to the underweight patients. This could be due to the fact that underweight patients may have had worse baseline health and less healthy food intake to have the energy to frequent the clinics and keep their appointments than patients who are normal weight. Additionally, the underweight patients may have had concomitant opportunistic infections that held them back from the clinics for replenishment of medicines and some may have died from such conditions, albeit from other health facilities. This finding was in agreement with other studies [9, 12, 29].

The hospital level was also associated with a lower likelihood for LTFU compared to health centre III level. Patients who received care from the hospital were 78% less likely to get LTFU, compared to those who went to health centre IIIs. Perhaps this is due to the difference in the quality of care offered at the hospital level compared to the lower level health facilities [30]. Additionally, there could be a lesser capacity by the lower facilities to manage the long queues, as affirmed by some patients in the in-depth interviews, that there were often long queues at the clinics and they had to wait for long hours. This makes patients reluctant to return to facility for ART replenishment. The long queues could, as well as, arise due to more patient loads, short intervals of appointment, among others [31, 32].

The qualitative findings revealed that transportation and long distance to the health facilities was a major challenge, and were common amongst the lost patients. An expert client revealed that some patients get to travel 15 to 20 km from home to the clinic for ART replenishment and care. A similar research also reported that patients who reside closer to the clinic were associated with better appointment attendance [33].

The conduct of the clinic staff was also critical in ensuring that patients were retained in care, and this involved professionalism of clinic staff and maintaining confidentiality of patients’ information. Our findings from in-depth interviews suggested that some patients were not impressed with the conduct of some clinic staff. Studies have also reported that having a positive relationship with the clinic staff was critical in ensuring patients stay at the clinic [34, 35].

Stigma was still a big challenge in the treatment and management of the patients. The findings from some key informants suggested that some patients refuse to be transferred to facilities nearer to them because of stigma, and hence they get to miss appointments on some days. Yehia et al. (2015) also reported that stigma was high amongst the non-retained patients [35].

The main limitation in our study was that patients initiated in the era of “test and treat” (between January 1st, 2017 and December 31st, 2017) who did not have documented CD4 cell count at baseline (almost two thirds of the “test and treat” population) were not included in the sampling frame. Patients missed baseline CD4 cell count (n = 7721) mainly because of the change to “test and treat” where clinicians didn’t have to know their CD4 cell count in order to initiate them into care and/or they may not have presented with symptoms. Thus, the patients who were included in the study from the “test and treat” strategy are likely to be less ill compared to those not recruited. Our study was also limited by the fact that we didn’t compare patients missing information to those not missing, on important characteristics that are related with LTFU, to assess for potential bias. These limitations may compromise on the external validity of the study. Additionally, the patient ART card was quite limited to enable other potential factors to be assessed in this study. Nonetheless, our study had sufficient power (sample size = 646 patients) to provide evidence on the problem of LTFU and its associated factors in the public health care setting.

## Conclusions

The incidence of LTFU in public health facilities in Uganda is quite high and is associated with being underweight, not having a documented telephone contact to receive reminders and receiving care at lower level facilities. Early diagnosis, routine use of patient address locator forms and improved quality of HIV care at lower level health facilities may reduce loss to follow up among PLHIV.

## Additional files

Additional file 1: In-depth Patient Interview Guide. Themes used to collect data during the in-depth interviews with patients. (PDF 530 kb)

Additional file 2: Key Informant Interview Guide. Themes used to collect data during the key informant interviews with health personnel. (PDF 518 kb)

## Data Availability

The datasets used and/or analyzed during the current study are available from the corresponding author on reasonable request.

## Abbreviations

ART: Anti-retroviral Therapy
BMI: Body Mass Index
CD4: Cluster of Differentiation 4
CI: Confidence Interval
eMTCT: Elimination of Mother-to-Child Transmission of HIV and Syphilis
HC: Health Centre
HCFs: Health Care Facilities
HIV: Human Immunodeficiency Virus
HIV/ AIDS: Human Immunodeficiency Virus/ Acquired Immune deficiency Syndrome
HR: Hazard Ratio
KI: Key Informant
KII: Key Informant Interview
LTFU: Loss to Follow- Up
MOH: Ministry of Health
PEP: Post-Exposure Prophylaxis
PLHIV: People Leaving with HIV
PrEP: Pre-Exposure Prophylaxis
STI: Sexually Transmitted Infections
TB: Tuberculosis
WHO: World Health Organization
