# Supplementary material for: Loss to follow-up and associated factors among adult people living with HIV at public health facilities in Wakiso district, Uganda: a retrospective cohort study
Source: BMC Health Serv Res. 2019 Sep 4;19:628. doi: 10.1186/s12913-019-4474-6 (PMC6727328; doi:10.1186/s12913-019-4474-6)
Supplement: Supplementary file 1 — In-depth Patient Interview Guide. Themes used to collect data during the in-depth interviews with patients. (PDF 530 kb) [file 12913_2019_4474_MOESM1_ESM.pdf]

BHSR-D-19-01067R2

‘Loss to follow-up and associated factors among adult people living with HIV at public health facilities in Wakiso district, Uganda: a retrospective cohort study’

Denis Opio, M.Sc.; Fred C. Semitala, MBChB, MMed, MPH-Epi, Imp Science, FCP(ECSA); Alex Kakeeto, B.Sc., M.Sc.; Emmanuel Sendaula, B.Sc., M.Sc.; Paul Okimat, B.Sc., M.Sc.; Brenda Nakafeero, B.Sc., M.Sc.; Joaniter I. Nankabirwa, MBChB, M.Sc., PhD; Charles Karamagi, MBChB, MMED, PhD; Joan N. Kalyango, B.Pharm, M.Sc., PhD

Additional file 1: In-depth Patient Interview Guide

Instructions: 1) Probe for how and why, where applicable and keep all questions open ended; 2) Specific probes are in Agency FB, in brackets.

Thematic area	Key questions	Methodology
**Waiting time**	• What do you think about the waiting time that patients have to spend on the queue while waiting for care at the clinic? (probe for the long waiting time, what is the adequate waiting time …)<br>• Can the length of waiting time lead to drop-out of a patient from care? (probe further for how …)	• Probing
**Social support**	• Do you have family/friends/relatives?<br>• How are these friends/relatives/family supportive in your healthcare? (probe further on how they are supported…)<br>• Can the support motivate a patient to keep in care? (probe further on how…)	• Probing<br>• Priority ranking<br>• Listing
**Stigma**	• Do you think stigmatization leads to drop out of patients from the health care? (probe further on how….)<br>• Have you ever been stigmatized due to your HIV status?<br>• If yes, in what different ways have you been	• Probing<br>• Priority ranking<br>• Listing

	stigmatized? (probe for the various forms of stigmatization…)<br>• How did you cope with the stigmatization? (probe for the various ways..)	
**Transportation**	• How does transportation lead to drop-out of patients from care? (probe for reliability of the transport means, the costs involved in transport and how long it takes for the patient to reach the ART clinic …)	• Probing<br>• Priority ranking<br>• Listing
**Patient- friendly services**	• Does the ART clinic provide schedules/reminders? (probe on how often, and when the reminders are done…)<br>• Do you think these reminders are helpful? (probe on how…)<br>• What do you think about the conduct of health workers at the ART clinic? (probe for privacy & confidentiality, ethical issues, professionalism…)<br>• Do you think the health workers' conduct may motivate patients to be retained in care? (probe on how…)	• Probing<br>• Priority ranking<br>• Listing
**Competing life activities**	• Do you have any activities you are engaged in? (probe for activities like employment, education…)<br>• Do you think these activities hinder retention in care? (probe on how…)	• Probing<br>• Priority ranking<br>• Listing
**Beliefs and misconceptions**	• Are there common beliefs/ or misconceptions regarding receiving HIV care in this community? (probe for the different kinds.)<br>• Do you think these beliefs can lead to drop out of a	• Probing<br>• Priority ranking<br>• Listing

	patient from care? (probe for reasons…)<br>• How have you perceived the different beliefs about receiving HIV care (probe for perceptions…)	
