# Supplementary material for: Loss to follow-up and associated factors among adult people living with HIV at public health facilities in Wakiso district, Uganda: a retrospective cohort study
Source: BMC Health Serv Res. 2019 Sep 4;19:628. doi: 10.1186/s12913-019-4474-6 (PMC6727328; doi:10.1186/s12913-019-4474-6)
Supplement: Supplementary file 2 — Key Informant Interview Guide. Themes used to collect data during the key informant interviews with health personnel. (PDF 518 kb) [file 12913_2019_4474_MOESM2_ESM.pdf]

BHSR-D-19-01067R2

'Loss to follow-up and associated factors among adult people living with HIV at public health facilities in Wakiso district, Uganda: a retrospective cohort study'

Denis Opio, M.Sc.; Fred C. Semitala, MBChB, MMed, MPH-Epi, Imp Science, FCP(ECSA); Alex Kakeeto, B.Sc., M.Sc.; Emmanuel Sendaula, B.Sc., M.Sc.; Paul Okimat, B.Sc., M.Sc.; Brenda Nakafeero, B.Sc., M.Sc.; Joaniter I. Nankabirwa, MBChB, M.Sc., PhD; Charles Karamagi, MBChB, MMED, PhD; Joan N. Kalyango, B.Pharm, M.Sc., PhD

Additional file 2: Key Informant Interview Guide

Instructions: 1) Probe for how and why, where applicable and keep all questions open ended; 2) Specific probes are in Agency FB, in brackets.

Thematic area	Key questions	Methodology
**Documentation**	• What do you think about the documentation of patient information in the ART clinic? (probe for the knowledge of documentation, adequacy, importance…)<br>• What needs to be done to improve the quality/ adequacy of documentation of patient information to improve retention in the ART clinics? (probe for the different strategies,)	• Probing
**Waiting time**	• What do you think about the waiting time the patients have to spend on the queue while waiting for care at the clinic? (probe for the long waiting time, how waiting time is affecting retention…)<br>• Do you think the length of waiting time influences the drop-out rate of patients from care? (probe further for how …)<br>• What are you doing to motivate the patients to keep coming for their medication? (probe for the different strategies.)	• Probing<br>• Priority ranking<br>• Listing

**Patient- friendly services**	• Does the ART clinic provide schedules/reminders to ensure patients are retained in care? (probe on how often, and when the reminders are done, influence on retention…)<br>• What do you think about the conduct of health workers at the ART clinic? (probe for privacy & confidentiality, ethical issues, professionalism…)<br>• Do you think the conduct of health workers may influence the patient to be retained in care? (probe on how it motivates, or demotivates retention…)	• Probing<br>• Priority ranking<br>• Listing
**Model of care**	• What do you think about the model of HIV care/refill venue of the health facilities? (probe for accessibility, different models of care …)<br>• Do you think the model of care may lead to patient drop-out? (probe for how, and why …)<br>• What are you doing about the model of care in order for the services to be more accessible to the patients? (probe further for how …)	• Probing<br>• Priority ranking<br>• Listing
